# Primary isolated hepatic tuberculosis mimicking small hepatocellular carcinoma: A case report

**DOI:** 10.1097/MD.0000000000022580

**Published:** 2020-10-09

**Authors:** Caiwei Yang, Xijiao Liu, Wenwu Ling, Bin Song, Fei Liu

**Affiliations:** a Department of Radiology; b Department of Ultrasonography; c Department of Liver Surgery, West China Hospital, Sichuan University, Chengdu, Sichuan Province, China.

**Keywords:** contrast-enhanced ultrasonography, hepatic, hepatocellular carcinoma, magnetic resonance imaging, tuberculosis

## Abstract

**Rationale:**

Mycobacterium tuberculosis (TB) remains a serious threat in developing countries. Primary isolated hepatic tuberculosis is extremely rare. Because of its non-specific imaging features, noninvasive preoperative imaging diagnosis of isolated hepatic tuberculoma remains challenging.

**Patient concerns:**

A 48-year-old man was admitted to our hospital due for suspected liver neoplasm during health examination.

**Diagnoses:**

The tests for blood, liver function, and tumor markers were within normal range. Preoperative ultrasonography (US) showed a hypoechoic lesion with a longitudinal diameter of 2.5 cm in segment six of liver. It exhibited early arterial phase hyperenhancement and late arterial phase rapid washout in contrast-enhanced US. It demonstrated hyperintensity in T2-weighted magnetic resonance imaging and partly restricted diffusion in diffusion-weighted imaging. For this nodule, the preoperative diagnosis was small hepatocellular carcinoma (HCC).

**Interventions:**

Laparoscopic hepatectomy was performed. Intraoperative extensive adhesion in the abdominal cavity and liver was found. The lesion had undergone expansive growth.

**Outcomes:**

Microscopically, a granuloma with some necrosis was detected. With both acid-fast staining and TB fragment polymerase chain reaction showing positive results, TB was the final histology diagnosis. After surgery, the patient declined any anti-TB medication. During the follow-up, he had no symptoms. In the sixth month after surgery, he underwent an upper abdominal US. It showed no lesions in the liver.

**Lessons:**

Because of non-specific imaging findings and non-specific symptoms, a diagnosis of isolated hepatic TB is difficult to make, especially for small lesions. A diagnosis of HCC should be made cautiously when small isolated lesions in the liver are encountered, especially in patients without a history of hepatitis and with negative tumor markers.

## 1 Introduction

Mycobacterium tuberculosis (TB) is and remains a growing public health threat worldwide, especially in developing countries.^[1]^ In terms of secondary disseminated tuberculosis, hepatic tuberculosis is seen in 50% to 80% of cases.^[2]^ However, isolated hepatic TB is a rare entity and poorly described in the literature, even in countries with a high prevalence of tuberculosis.^[3]^ Patients always have non-specific clinical findings. Isolated hepatic TB responds well to anti-tubercular treatment.

Imaging plays an important role in detection of isolated hepatic TB detection. However, due to non-specific imaging manifestation, hepatic TB may cause a diagnostic dilemma.^[4]^ It may be misdiagnosed as a liver tumor, such as intrahepatic carcinoma or metastasis, or other lesions, such as liver abscess or hydatid cyst and so on.^[5–9]^ Here, we report a case of isolated hepatic TB with primary misdiagnosis as small hepatocellular carcinoma (HCC) on contrast-enhanced ultrasonography (US) and magnetic resonance imaging (MRI). To our knowledge, similar cases have not been of such a small size.^[4–6]^ Furthermore, this is the first report on contrast-enhanced US findings of hepatic isolated TB. We discuss the findings of noninvasive imaging modalities of hepatic TB.

## 2 Case presentation

**Figure 1:**
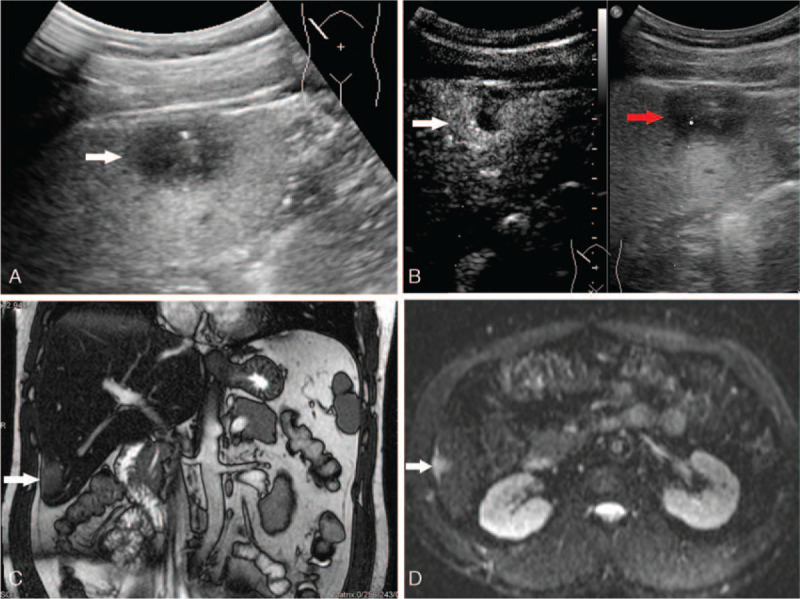
A: A hypo echoic lesion with a size of 2.5 cm x 1.8 cm located in the sixth segment of liver was detected on the transabdominal US. B: The lesion showed hyper enhancement on early arterial phase (white arrow) and quickly wash-out on late arterial phase (red arrow) after intravenous injection of sulfur hexafluoride-filled micro bubble contrast agent. C: A hyper intensity lesion with a size of 2.1 cm x 1.7 cm on T2-weight image of MRI was presented. D: The hyper intensity lesion was partly diffusion restricted on diffusion-weighted MR images.

A 48-year-old man was admitted to West China Hospital of SiChuan university for suspected liver neoplasm during health examination. He denied having a fever, weight loss, changes in appetite, or weakness. Clinical examination came back normal, and he denied any medical history of hepatitis or tuberculosis. The laboratory tests for blood and liver function were normal. The serum tumor markers (α-fetoprotein, carcinoembryonic antigen, CA-19.9, CA-125) were within the normal range. A plain chest radiography showed no evidence of pulmonary lesions. The patient underwent a transabdominal US, which demonstrated a hypoechoic lesion with a size of 2.5 cm x 1.8 cm, located in the sixth segment of the liver (Fig. 1A). After intravenous injection of sulfur hexafluoride-filled microbubble contrast agent, it showed early arterial phase hyperenhancement on (Fig. 1B white arrow) and late arterial phase rapid washout (Fig. 1B red arrow). Consequently, the patient underwent abdominal MRI, which showed a hyperintense nodule with a size of 2.1 cm x 1.7 cm on T2-weighted MRI (Fig. 1C) and partly restricted diffusion in diffusion-weighted imaging (Fig. 1D). After injection of extracellular contrast agents, the lesion manifested non-rim arterial phase hyperenhancement and presented portal venous phase washout. There was no enlarged lymph node or other lesion in the abdomen. Both US and MRI findings supported a HCC tumor.

**Figure 2:**
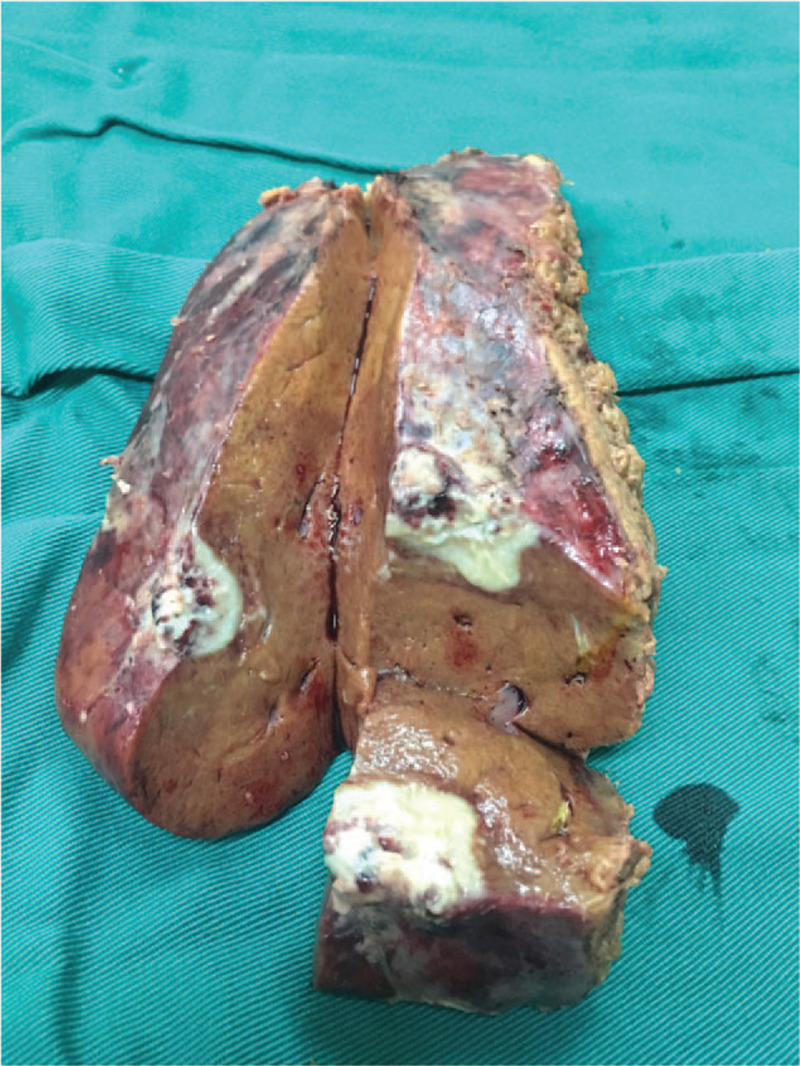
The lesion was fish like and irregular in the general pathology after surgical excision.

**Figure 3:**
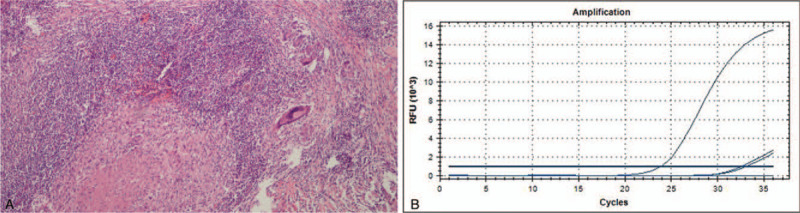
A: Histological examination detected granuloma with some necrosis (HE × 200). B: TB fragment polymerase chain reaction showed positive results.

Although a CT scan-guided biopsy may have been helpful in establishing a diagnosis, the patient denied the procedure and elected to undergo surgical excision. Then, a laparoscopic hepatectomy was performed. Intraoperatively there were no ascites found in the abdominal cavity: there was extensive adhesion among the omentum and peritoneum and liver; the hepatic Glisson's capsule was tense, liver margin was round, liver size was normal, and hepatic parenchyma had manifested moderate fatty liver change; the gallbladder was slightly congested, without adhering to the surrounding tissues. The nodule was in the sixth segment of the liver, exhibiting exophytic growth, adhering with the diaphragm. The tumor presented with a fish-like appearance and had irregular boundaries (Fig. 2). Microscopically, a granuloma with some necrosis was detected (Fig. 3A). With both acid-fast staining and TB fragment polymerase chain reaction showing positive results (Fig. 3B), TB was the final histology diagnosis. The patient then underwent the Mantoux test and the result was negative. For 1 year, the patient underwent follow-up. After surgery, he declined any anti-TB medication. During follow-up, he had no symptoms. In the sixth months after surgery, he underwent an upper abdominal US. It showed no liver lesions.

## 3 Discussion

TB persists globally. Hepatic TB's incidence has increased due to the acquired immunodeficiency syndrome epidemic, intravenous drug abuse, multidrug resistance, etc.^[10]^ Hepatic TB makes up 3% of all extra-pulmonary locations and 9% of intra-abdominal locations. Levine classified^[11]^ hepatic TB into:

(1) miliary TB;
(2) pulmonary TB with hepatic involvement;
(3) primary liver TB;
(4) focal tuberculoma or abscess;
(5) tuberculous cholangitis.

Miliary TB is the most common form of hepatic TB. Isolated hepatic TB is seldom encountered in clinical practice, with only a few sporadic cases and short series available in the current literature.^[12–15]^ We reported a case of isolated hepatic TB with a long diameter of 2.5 cm. As far as we know, this is the smallest primary isolated hepatic macronodular TB ever reported.

Hepatic TB's clinical presentation is usually silent and it is often incidentally encountered when the patient is being evaluated for mostly non-specific symptomatology. Laboratory tests may indicate impaired liver function, commonly due to cholestasis and cytolysis in the presentation of elevated hepatic enzymes. The sensitivity of serological tests for acid-fast staining bacilli and blood cultures is, respectively, as low as 0%–45% and 10%–60%.^[16]^ A tuberculin skin test, which is typically positive, and PCR have a sensitivity and specificity of 58% and 96%, respectively, and when used in combination improve the rate of detection.^[9,16]^ In the event of isolated elevation of alkaline phosphatase, the possibility of tubercular hepatic parenchymal involvement must be excluded.^[17–19]^ In our case, the patient had no clinical symptoms and the laboratory tests for blood and liver function were normal.

Imaging, including US, CT, and MRI, plays an important role in hepatic lesion detection and diagnosis. According to radio-pathological correlation, Yu et al classified hepatic tuberculosis into three subgroups: parenchymal, serohepatic and tubercular cholangitis.^[15]^ Parenchymal lesions are further subclassified into patterns that are micronodular (<2 cm), macronodular (≥2 cm), and mixed micronodular–macronodular.^[20,21]^ Below, we focus on the imaging features of hepatic parenchymal TB.

Miliary TB is the most common form of hepatic parenchymal TB. It may present as multiple small nodules, with a diameter of approximately less than two cm and tend to be randomly dispersed across the whole hepatic parenchyma. In US imaging, these lesions appear hypoechoic to isoechoic compared to the liver background.^[12–14,22–24]^ In plain CT imaging, miliary lesions appear as micro abscesses in the form of multiple small foci with a low attenuation. The lesions may exhibit peripheral enhancement, which is difficult to differentiate from metastases, lymphoma, or other forms of granulomatous disease.^[5,15]^

In terms of primary isolated hepatic parenchymal TB, in US images, the lesion can range from being heterogeneously hypoechoic, mixed hypoechoic to hyperechoic. Our patient underwent contrast-enhanced US. After intravenous injection of sulfur hexafluoride-filled microbubble contrast agent, the nodule showed early arterial phase hyperenhancement and late arterial phase rapid washout. This feature led to a misdiagnosis of tumor. As far as we know, this is the first report on contrast-enhanced US findings of isolated hepatic TB.

CT imaging features of isolated hepatic TB vary in the different stages of the disease. Hepatic tuberculoma displays hypodensity on plain imaging and often shows barely or slightly peripheral rim enhancement. As a result, such a lesion present in isolation may create a diagnostic dilemma, making it practically impossible to differentiate from hepatic metastasis or other malignant tumors. In imaging, the appearance of tubercular abscesses with frank caseous necrosis change with the degree and distribution of internal liquefaction necrosis.^[10,12,15]^ In abdominal CT scans, tuberculoma with central low-density areas caused by caseating necrosis usually presents as a central non-enhancing lesion with a peripheral enhancing rim of outer granulation tissue. Rarely, the lesions can present with extensive necrosis being thus similar to cysts manifesting no discernible peripheral enhancement.^[15,20,25]^ Additionally, various kinds of calcification patterns on the lesions can be seen in CT. The incidence of calcification ranges from 0% to 64%.^[15,26,27]^ Vascular complications, such as portal vein thrombosis and subsequent portal hypertension, have also been reported.^[28]^

MRI remains a valuable noninvasive imaging modality for the detection and differentiation of hepatic tuberculoma. For the diagnosis of hepatic TB, the accuracy of MRI is higher than that of CT.^[15,28]^ The nodular tubercular lesions exhibit hypointensity in T1-weighted images and varied intensity with a peripherally hypointense rim in T2-weighted images. In dynamic phase, they may display rim or heterogeneous enhancement.^[14,15,29]^ The nodules may show slightly restricted diffusion in diffusion-weighted images.

The diagnosis in our case was difficult due to non-specific symptoms and variations of the above non-specific imaging findings. Considering the non-rim enhancement pattern and the restricted diffusion features, both contrast-enhanced US and MRI were likely to lead to a diagnosis of tumor of HCC. However, this patient did not have liver cirrhosis or a history of hepatitis. Moreover, the serum tumor markers (α-fetoprotein, carcinoembryonic antigen, CA-19.9, CA-125) were normal. The present case reminds us to make a diagnosis of HCC cautiously when encountering small isolated liver lesions, especially in patients without a history of hepatitis and with negative tumor markers.

Generally, imaging plays a valuable role in the detection of tubercular hepatic lesions. Additionally, imaging could be helpful in their differential diagnosis and for assessing associated complications. However, these findings are not always so specific,^[15]^ and a histopathological or bacteriological confirmation is required as a practicality. Concomitant disease elsewhere, such as nodal or pulmonary involvement, can be a useful clue to narrowing down the list of differential diagnoses. And yet, primary isolated hepatic TB poses a real diagnostic imaging dilemma in the absence of the above concomitant diseases, let alone when it presents as a primary, small-sized hepatic TB mimicking a tumor. The present case reminds us to make a diagnosis of HCC cautiously when encountering small isolated liver lesions, especially in patients without a history of hepatitis and with negative tumor markers.

## Author contributions

**Conceptualization:** Bin Song, Fei Liu.

**Data curation:** Caiwei Yang, Wenwu Ling.

**Writing – original draft:** Caiwei Yang, Xijiao Liu.

**Writing – review & editing:** Caiwei Yang, Xijiao Liu, Wenwu Ling, Bin Song, Fei Liu.

## Abbreviations

Abbreviations: AIDS = the acquired immunodeficiency syndrome, HCC = hepatocellular carcinoma, MRI = magnetic resonance imaging, TB = tuberculosis, US = ultrasonography.
